# Cytotoxic T lymphocyte antigen-4 expression in esophageal carcinoma: implications for prognosis

**DOI:** 10.18632/oncotarget.8476

**Published:** 2016-03-30

**Authors:** Xiao-Fei Zhang, Ke Pan, De-Sheng Weng, Chang-Long Chen, Qi-Jing Wang, Jing-Jing Zhao, Qiu-Zhong Pan, Qing Liu, Shan-Shan Jiang, Yong-Qiang Li, Hong-Xia Zhang, Jian-Chuan Xia

**Affiliations:** ^1^ State Key Laboratory of Oncology in Southern China, Collaborative Innovation Center for Cancer Medicine, Sun Yat-sen University Cancer Center, Guangzhou 510060, People's Republic of China; ^2^ Department of Biotherapy, Sun Yat-sen University Cancer Center, Guangzhou 510060, People's Republic of China

**Keywords:** esophageal carcinoma, overall survival, cytotoxic T lymphocyte antigen-4

## Abstract

To examine the relationship between cytotoxic T lymphocyte antigen-4 (CTLA-4) expression and esophageal carcinoma prognosis, CTLA-4 expression was immunohistochemically detected in paraffin-embedded primary tumor specimens from 158 patients with esophageal cancer. CTLA-4 was detected in the cytoplasm and cell membranes of esophageal cancer cells and in interstitial lymphocytes. In univariate analyses (log-rank), higher interstitial CTLA-4^+^ lymphocyte density and higher tumor CTLA-4 expression were associated with shorter overall survival (OS). After controlling for age and clinical stage, multivariate analysis (Cox) found that tumor CTLA-4 expression was an independent predictor of shorter OS (HR 2.016, *P* = 0.004). These results indicate that CTLA-4 expression in the tumor environment (both lymphocytes and tumor cells) is associated with poorer prognosis. In addition, CTLA-4 profiles may be useful for predicting the benefits and toxicity of CTLA-4 blockade in patients with esophageal carcinoma.

## INTRODUCTION

Esophageal cancer is a serious malignancy with poor prognosis and high mortality rate [1–3]. It is the eighth most common cancer, and the sixth most common cause of cancer-related deaths worldwide, with more than 80% of all cases and deaths occurring in developing nations [4]. In 2012, approximately 400,000 deaths due to esophageal cancer were reported, accounting for approximately 5% of all cancer deaths. In addition, approximately 456,000 new cases were diagnosed, representing 3% of all cancers [5]. While the incidence of other types of cancers is expected to decrease over the next 10 years, by 2025 the prevalence of esophageal cancer is expected to increase by 140% [6].

Despite many advances in diagnosis and treatment, the 5-year survival rate for patients with esophageal cancer ranges from 15% to 20% [7]. Moreover, a majority of patients (60%–70%) do not respond well to neoadjuvant regimens and develop severe adverse effects [8,9]. Thus, to develop novel therapeutic strategies and improve patient prognosis, we must elucidate the mechanisms underlying esophageal cancer pathogenesis.

Tumor-derived immune dysregulation is a key feature of esophageal cancer. Cancer proteomics studies have identified diagnostic, prognostic and predictive biomarkers which can be used for early cancer detection and prediction of the clinical behavior of the disease, as well as for the identification of novel molecular targets involved in tumorigenesis and disease progression. Several additional underlying molecular mechanisms have been discovered (including genetic alteration, growth factors, and angiogenesis) and have been the basis for a number of potential therapies for esophageal cancer [10–13]. In addition, the immunosuppressive microenvironment derived from esophageal cancer cells, consisting of cytokines and immune checkpoint molecules, may also be involved in tumor growth and metastasis in esophageal cancer [14–16].

Cytotoxic T lymphocyte antigen-4 (CTLA-4, CD152) is an immune checkpoint molecule and a CD28 homologue that binds the ligands B7-1 (CD80) and B7-2 (CD86) [17]. Human CTLA-4 has two different isoforms: a full-length membrane-bound receptor isoform (mCTLA-4) with an extracellular ligand-binding domain and an intracellular signal-transducing domain, and a secreted, soluble isoform (sCTLA-4), which consists of only the extracellular domain [18,19].

Although CD28 is highly expressed on the surface of resting T cells, CTLA-4 is localized intracellularly within clathrin-associated complexes [20]. Stimulation of naïve T cells through the T cell receptor causes rapid and transient translocation of intracellular CTLA-4 to the cell surface, or its extracellular secretion [21–23]. The two different CTLA-4 isoforms reduce T cell activation (both intrinsically and extrinsically) by forming a negative feedback loop to maintain immune self-tolerance and homeostasis. CTLA-4 outcompetes CD28 for B7 ligands, attenuating the effector T cell response through the inhibition of IL-2 and blockade of cell cycle progression [24].

Constitutive CTLA-4 expression on T regulatory cells (Tregs) reduces the level of B7 ligand on antigen presenting cells, further inhibiting effector T cell immunity [25]. In addition, CTLA-4-expressing cells trans-endocytose ligands on neighboring cells, preventing CD28 co-stimulation [26]. Soluble CTLA-4 also interacts with B7, inhibiting T cell activity by interfering with CD28 signaling, and blocking soluble CTLA-4 enhances antigen-driven peripheral blood mononuclear cell responses [23]. Although CTLA-4 expression by T cells during acute antigen exposure is transient, chronic antigen exposure, as in cancer, leads to sustained expression of CTLA-4 [27].

CTLA-4 has been implicated in immune dysregulation of B cell chronic lymphocytic leukemia [28], non-Hodgkin's lymphoma [29], breast cancer [30], lung cancer [31, 32], skin cancer [33, 34], gastric cancer [35, 36], colorectal cancer [37, 38] and cervical cancer [39–41]. Furthermore, CTLA-4 protein expression in cancer appears to be important for tumors to evade host immune surveillance. However, the clinical implications of CTLA-4 expression in tumors or immune cells in the tumor microenvironment are still controversial, and the potential for CTLA-4 as a prognostic marker has been complicated by differences in study populations and methods. Furthermore, there is no established functional or causal relationship between CTLA-4 expression in tumors and immune cells in the tumor microenvironment, or in patient prognosis, in esophageal cancer.

## RESULTS

### CTLA-4 expression on tumor cells or TIMCs

Patient and tumor characteristics are shown in Supplementary Table S1. Tumor samples were obtained from 158 patients with adequate clinical data for evaluation of CTLA-4 expression in tumor cells. Among these samples, 154 had TIMCs that were evaluable for CTLA-4 expression.

**Figure 1:**
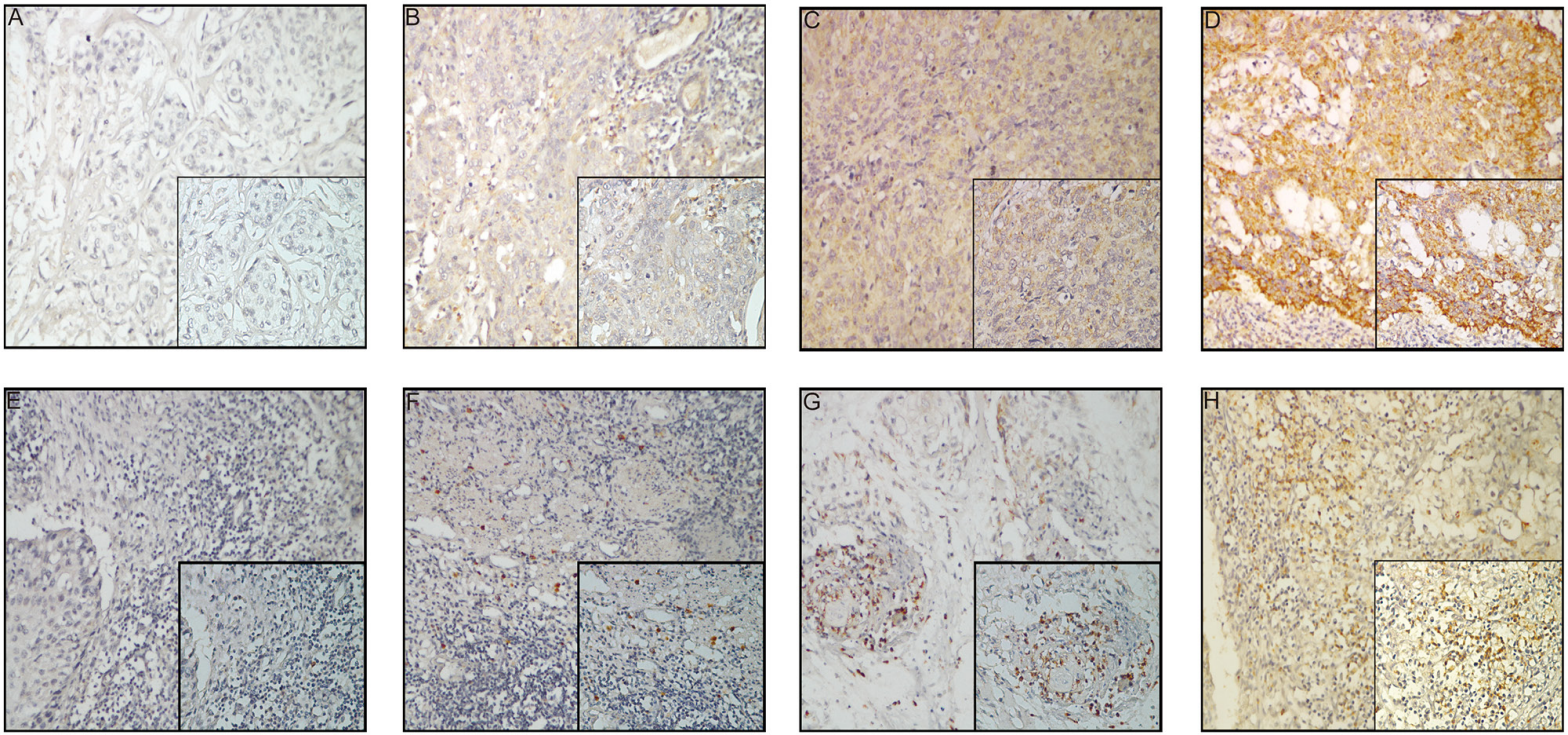
CTLA-4 expression in esophageal carcinoma samples **A–D.** Tumor cells: (A) negative CTLA-4 expression; (B) weak CTLA-4 expression; (C) moderate CTLA-4 expression; (D) strong CTLA-4 expression; 200× magnification, Inset: 400× magnification; **E–H.** TIMCs: (E) focal CTLA-4 expression; (F) mild CTLA-4 expression; (G) moderate CTLA-4 expression; (H) severe CTLA-4 expression; 200× magnification, Inset: 400× magnification.

CTLA-4 was expressed in the cell membrane, cytoplasm, or both, either in a focal or scattered pattern (Figure 1). For the vast majority of ESCC cases, CTLA-4-positive cells were scattered evenly throughout the specimen, in a similar form to that observed in glioma [43] and ovarian cancer [44]. CTLA-4 expression was observed in 87% (137/158) of the cases (Figure 1A-D). Elevated CTLA-4 expression (“+” and “++”) was detected in 52.6% (72/137) of samples expressing CLTA-4 (Supplementary Table S2).

Of the 154 patients with TIMCs, CTLA-4 expression in TIMCs was scored as absent [0] in 23 patients (14.9%), focal [1] in 42 patients (27.3%), mild [2] in 53 patients (34.4%), moderate [3] in 20 patients (13.0%) and severe [4] in 16 patients (10.4%; Figure 1E-H). CTLA-4 expression in TIMCs was negative (score of 0 or 1) in 65/154 patients (42.2%) and positive [2–4] in 89/154 patients (57.8%) (Supplementary Table S3).

### Correlation between CTLA-4 expression and postoperative prognosis

**Figure 2:**
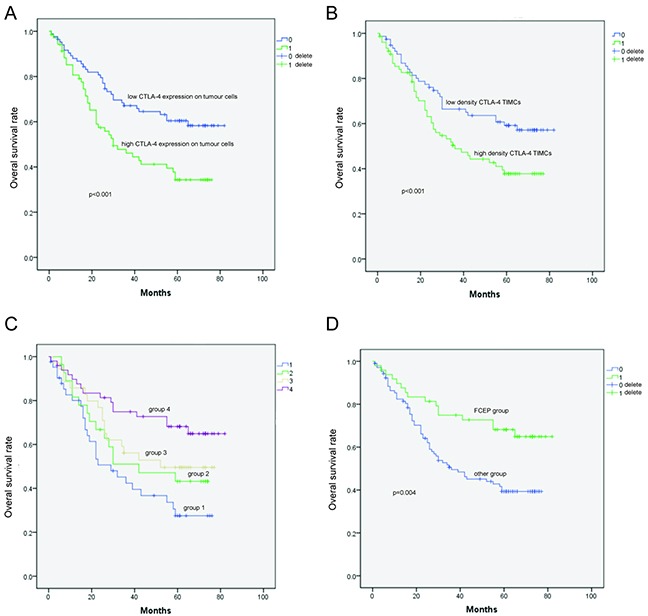
Kaplan–Meier survival curves showing the correlation between CTLA-4 expression and prognosis **A.** Overall survival (OS) of patients with high CTLA-4 expression in tumor cells was shorter than that of patients with low CTLA-4 expression in tumor cells. **B.** OS of patients with high-density CTLA-4^+^ interstitial lymphocytes was shorter than that of patients with low-density CTLA-4^+^ interstitial lymphocytes. **C.** OS of patients in Group 4 was longer than that of patients in Groups 1, 2, and 3. **D.** Low CTLA-4 expression in tumor cells and low-density CTLA-4^+^ interstitial lymphocytes was designated as the favorable CTLA-4 expression profile (FCEP) group; the other patients were designated as the “Other patients” group. OS of patients in the FCEP group was longer than that of the “Other patients” group.

We next examined the relationship between CTLA-4 expression and various prognostic factors. There was no relationship between CTLA-4 expression (either in tumor cells or TIMCs) and age at the time of surgery, sex, tumor (T), nodal (N) or metastatic (M) status, or pathologic stage (Supplementary Tables S2 and S3). Interestingly, the OS of CTLA-4-positive patients was worse than that of CTLA-4-negative patients (36 vs. 65 months, *P* < 0.001; Figure 2A) in both univariate (*P* = 0.003) and multivariate analyses (*P* =0.004) (Table 1). Positive CTLA-4 expression (score of 2–4) in TIMCs was associated with shorter OS (38 vs. 64 months, *P* < 0.001; Figure 2B) in univariate analyses (P = 0.018; Table 2). However, there was no correlation found between CTLA-4 expression in tumor cells and interstitial lymphocytes.

**Table 1:** Univariate and multivariate analyses of overall survival in esophageal carcinoma (Tumor CTLA-4 expression)

Variable	Univariate analyses			Multivariate analyses		
	HR	95% CI	*P*-value	HR	95% CI	*P*-value
Tumor CTLA-4 expression	1.977	1.251–3.125	0.003^a^	2.016	1.249–3.253	0.004^a^
Histological grade	1.701	1.229–2.356	0.001^a^	1.460	1.028–2.704	0.034^a^
Age	1.101	0.700–1.734	0.677	1.101	0.672–1.804	0.702
Sex				1.269	0.675–2.387	0.460
Length	1.179	0.741–1.877	0.488	1.054	0.628–1.772	0.842
Location	1.153	0.774–1.718	0.483	1.077	0.721–1.609	0.717
T	2.368	1.323–4.237	0.004^a^	1.969	0.920–4.213	0.081
N	2.811	1.766–4.474	<0.001^a^	2.110	0.864–5.153	0.101
M	2.312	0.933–5.731	0.07	1.484	0.466–4.725	0.504
TNM	3.368	2.130–5.324	<0.001^a^	1.340	0.503–3.574	0.558

Abbreviations: HR, hazard ratio; CI, confidence interval; TNM, tumor, node, metastasis.

a *P* < 0.05.

**Table 2:** Univariate and multivariate analyses of overall survival in esophageal carcinoma (density of CTLA-4^+^ TIMCs)

Variable	Univariate analyses			Multivariate analyses		
	HR	95% CI	*P*-value	HR	95% CI	*P*-value
Density of CTLA-4 +TIMCs	1.744	1.099–2.767	0.018^a^	1.472	0.913–2.374	0.113
Histological grade	1.701	1.229–2.356	0.001^a^	1.460	1.025–2.708	0.036^a^
Age	1.101	0.700–1.734	0.677	1.101	0.672–1.804	0.702
Sex				1.184	0.625–2.244	0.605
Length	1.179	0.741–1.877	0.488	1.136	0.666–1.938	0.639
Location	1.153	0.774–1.718	0.483	1.185	0.802–1.751	0.395
T	2.368	1.323–4.237	0.004^a^	1.845	0.853–3.990	0.119
N	2.811	1.766–4.474	<0.001^a^	1.770	0.729–4.292	0.207
M	2.312	0.933–5.731	0.07	1.560	0.485–5.016	0.456
TNM	3.368	2.130–5.324	<0.001^a^	1.340	0.503–3.574	0.558

Abbreviations: HR, hazard ratio; CI, confidence interval; TIMCs: tumour-infiltrating mononuclear cells; TNM, tumor, node, metastasis.

a *P* < 0.05.

### Correlation of survival with tumor cell CTLA-4 expression and CTLA-4^+^ TIMC density

Patients were divided into four subgroups according to their profiles of tumor cell CTLA-4 expression and CTLA-4^+^ TIMC density: Group 1 (CTLA-4^high^ tumor cells, density^high^ CTLA-4^+^ TIMCs), Group 2 (CTLA-4^high^ tumor cells, density^low^CTLA-4^+^ TIMCs), Group 3 (CTLA-4^low^ tumor cells, density^high^CTLA-4^+^ TIMCs), and Group 4 (CTLA-4^low^tumor cells, density^low^CTLA-4^+^ TIMCs). Univariate analysis (log-rank) revealed that mean OS was longer in Group 4 (62.705 months, Table 3; *P* = 0.009, Table 4) than in Group 1 (43.822 months, Table 3; *P* = 0.005, Table 4), Group 2 (45.702 months, Table 3; *P* = 0.001, Table 4) and Group 3 (49.712 months; Table 3; *P* = 0.007, Table 4), indicating that higher intratumoral CTLA-4 expression was associated with increased risk of death from ESCC (Figure 2C).

**Table 3:** Overall survival (OS) of groups divided by CTLA-4 expression profiles

Groups	Tumor CTLA-4 expression	Density of CTLA-4^+^ TIMCs	Number	Mean OS (month)	95% CI
Group 1	High	High	42	43.822	29.041–46.602
Group 2	High	Low	28	45.702	34.167–55.238
Group 3	Low	High	35	49.712	40.068–59.356
Group 4	Low	Low	49	62.705	54.495–70.915

Abbreviations: HR, hazard ratio; CI, confidence interval; OS, overall survival; TIMCs: tumour-infiltrating mononuclear cells; a: *P* < 0.05.

**Table 4:** Univariate of overall survival in esophageal carcinoma (groups)

Variable	Univariate analyses		
	HR	95% CI	*P*-value
Group 1	1.452	0.260-0.787	0.005^a^
Group 2	1.701	1.229-2.356	0.001^a^
Group 3	1.101	0.700-1.734	0.007^a^
Group 4	0.954	0.541-1.680	0.009^a^

Abbreviations: HR, hazard ratio; CI, confidence interval.

a *P* < 0.05.

### Correlation between favorable CTLA-4 expression, clinical characteristics, and prognosis

Based on the aforementioned analysis, Group 4 was designated as the “favourable CTLA-4 expression profile” (FCEP) Group, and patients in Groups 2, 3 and 4 were designated as the “Other patients” group. No differences in clinical features were detected between the two groups (Supplementary Table S4). However, univariate analysis (log-rank) showed that the OS of the FCEP group (*n* = 49, events = 16, mean OS = 62.705 months) was longer than that of the “Other patients” group (*n* = 105, events = 59, mean OS = 44.246 months; *P* = 0.004; Figure 2D). Multivariate analysis revealed that FCEP status independently predicted longer OS (HR 0.460, 95% CI 0.260–0.813, *P* = 0.008; Table 5).

**Table 5:** Univariate and multivariate analyses of overall survival in esophageal carcinoma (FCEP status)

Variable	Univariate analyses			Multivariate analyses		
	HR	95% CI	*P*-value	HR	95% CI	*P*-value
FCEP status	0.452	0.260–0.787	0.005^a^	0.460	0.260–0.813	0.008^a^
Histological grade	1.701	1.229–2.356	0.001^a^	1.508	1.050–2.166	0.026^a^
Age	1.101	0.700–1.734	0.677	1.062	0.639–1.765	0.817
Sex	0.954	0.541–1.680	0.869	1.262	0.668–2.383	0.473
Length	1.179	0.741–1.877	0.488	1.161	0.683–1.975	0.582
Location	1.153	0.774–1.718	0.483	1.123	0.759–1.662	0.560
T	2.368	1.323–4.237	0.004^a^	1.878	0.860–4.097	0.114
N	2.811	1.766–4.474	<0.001^a^	2.052	0.830–5.072	0.119
M	2.312	0.933–5.731	0.07	1.746	0.539–5.657	0.353
TNM	3.368	2.130–5.324	<0.001^a^	1.294	0.475–3.522	0.614

Abbreviations: HR, hazard ratio; CI, confidence interval; FCEP, favourable CTLA-4 expression profile; TNM, tumor, node, metastasis.

a *P* < 0.05.

## DISCUSSION

To our knowledge, this is the first study to demonstrate that elevated CTLA-4 expression in ESCC is associated with poor prognosis and that CTLA-4 expression in TIMCs is associated with esophageal cancer aggressiveness and shorter OS. Our study provides evidence that CTLA-4 promotes cancer progression, perhaps through impairment of host T cell-mediated immunity as has recently been reported [42].

CTLA-4 is a cell surface glycoprotein belonging to the B7 family of co-stimulatory molecules. Constitutive CTLA-4 expression is normally restricted to Treg cells, where it participates in the co-stimulatory activation of naïve T cells or depletion of activated T cells [1, 23]. However, CTLA-4 protein expression can be stimulated in TIMCs and tumor cells. Activation of naïve T cells (following specific antigen recognition) induces the expression of cytokines such as interferon-γ, which in turn induces CTLA-4 expression on surrounding immune and tumor cells. Tumor-associated CTLA-4 has been shown to inhibit anti-tumor T cell immunity by interacting with CD28 expressed on T cells to induce tumor-specific T cell apoptosis or by impairing cytokine production and T cell-mediated cytotoxicity [43]. CTLA-4 inhibits T cell immunity, and CTLA-4 blockade reverses this process [44–46]. Thus, CTLA-4 may function in the periphery as a negative regulator of effector T cell-mediated anti-tumor immunity, thereby allowing unrestrained tumor progression due to impaired host immune surveillance.

In accordance with these observations, we demonstrate that ESCC cells are capable of expressing CTLA-4. In addition, our analyses reveal that elevated tumor cell CTLA-4 expression is associated with shorter OS, and that increased CTLA-4^+^ TIMC density also increases the risk of death (relative risk of 3.58). Moreover, the combination of increased tumor cell CTLA-4 and/or high CTLA-4^+^ TIMC density is an even stronger predictor of patient outcome (relative risk of 4.53). We also found that regional lymph node, distant metastases, and histological grade are predictive of a poor prognosis (Table 5). Even after adjusting for each of these features, the association of intratumoral CTLA-4 expression with OS persisted.

Our observation that intratumoral CTLA-4 might facilitate ESCC progression and diminish patient survival has important implications for the immunobiology and immunotherapeutic treatment of ESCC tumors. For instance, several studies have reported defective anti-tumor immunity in ESCC patients. Such defects in immunity can be partly ascribed to upregulated intratumoral expression of immunosuppressive IDO [46], TGF-β1 [48], COX-2, VEGF, IL-8 [49], CCL17 and CCL22 [50]. In addition, ESCC patients have diminished responses to recall antigens [51], decreased proliferative T cell responses and cytokine production [52, 53], and defects in signal transduction between T cells and natural killer cells. Tumor-infiltrating T lymphocytes, including CD8^+^ and CD4^+^ T cells, are considered to be a manifestation of the host immune response in ESCC [54–57]. However, the clinical significance of each T cell subset in ESCC is still controversial. Thus, we speculated that CTLA-4 expressed by either ESCC tumor cells or infiltrating lymphocytes contributes to the profile of immunosuppression that is observed in ESCC patients based on its ability to impair the function and survival of activated tumor-specific T cells. An improved understanding of the biology of CTLA-4 expression in tumors is urgently required to identify effective manipulations for the improvement of current forms of immunotherapy.

Cellular and murine models have been used to demonstrate that CTLA-4 blockade augments endogenous responses to several tumor types, leading to tumor cell death when utilized alone or in combination other therapeutic interventions. Preclinical findings have translated into the clinical development of a fully human, IgG1 monoclonal antibody (mAb), ipilimumab (formerly MDX-010 or BMS-734016; Yervoy™, Bristol-Myers Squibb, Princeton, NJ, USA) and a fully human, IgG2 mAb, tremelimumab (formerly ticilimumab; CP-675,206, Pfizer, New York, NY, USA), both of which bind CTLA-4. Thus, antibody-mediated blockade of CTLA-4 may ultimately prove useful, either alone or in combination with other immune-based manipulations, to improve the effectiveness of ESCC treatment. In addition, CTLA-4 may serve as a predictive biomarker for selecting the most appropriate therapy for ESCC patients and maximizing the clinical benefit with minimal toxicity. However, although aberrant CTLA-4 expression is strongly implicated in immune dysfunction in ESCC, it is likely that multiple other host factors also contribute. Other immunosuppressive co-stimulatory molecules, including PD-L1, PD-L2 and regulatory T cells such as CD4^+^CD25^+^T cells, remain under investigation in the context of ESCC and may similarly facilitate the downregulation of anti-tumoral T cell responses.

## CONCLUSIONS

We found CTLA-4 expression in primary esophageal cancer lesions to have potential prognostic value, with higher CTLA-4 expression and higher density of interstitial CTLA-4^+^ lymphocytes associated with poorer prognosis. Analysis of CTLA-4 expression profiles in lymphocytes and tumor cells revealed marked variation among esophageal cancer patients. We speculate that these immunological features might be associated with clinical efficacy and adverse reactions to CTLA-4 blockade, and may help to guide immunotherapeutic strategies in the future. These findings suggest that further studies of immunotherapies guided by individual variation in the immune status of patients are warranted.

## MATERIALS AND METHODS

### Patients

A total of 158 paraffin-embedded esophageal squamous cell carcinoma (ESCC) samples were obtained from patients who underwent surgery at the Sun Yat-sen University Cancer Center between 2002 and 2003. There were 126 male and 32 female patients with a median age of 56 years (range, 33–78 years). Patients with autoimmune diseases and other kinds of esophageal cancer (e.g., adenocarcinoma) were excluded. Before surgical resection, none of the patients had received any anti-cancer treatment. Histological cell types of tumor tissues were classified according to World Health Organization criteria. There were 100 cases of stage I–II and 58 cases of stage III–IV cancer according to the American Joint Committee on Cancer (AJCC, 2002) TNM staging system. The follow-up data from the ESCC patients involved in this study are available and complete. The postoperative follow-up was carried out in our outpatient department and included regular clinical and laboratory examinations as follows: every 3 months for the first 2 years, every 6 months for the following 2 years, and annually for an additional 5 years or until patient death, whichever occurred first. This study was approved by the Ethics Committee of Sun Yat-sen University Cancer Center, and informed consent was obtained from each patient.

### Immunohistochemistry

Serial paraffin-embedded sections (2 μm thick) from the 158 patients were de-waxed with xylene and subsequently hydrated with an ethanol gradient. For antigen retrieval, the tissue sections were immersed in EDTA (1 mmol/l, pH 9.0) and maintained at 100°C for 15 minutes, before cooling at room temperature for 2 h. The sections were then washed with phosphate-buffered saline (PBS, pH 7.4) and immersed in 3% H_2_O_2_ for 15 min to eliminate endogenous peroxidase activity. After incubation in 10% normal goat serum (Invitrogen, Paisley, UK) for 30 min at room temperature to block non-specific antigens, sections were then incubated overnight at 4°C with the primary detection antibody (monoclonal mouse anti-human CTLA-4, Abcam, US, ab134090, 1:500 dilution). Excess antibody was removed by gentle rinsing, and the sections were washed with PBS three times. Subsequently, the sections were incubated with horseradish peroxidase-conjugated secondary antibody (EnVision™ Detection Kit, GK500705, Gene Tech) at room temperature for 30 min. After washing three times with PBS, sections were stained with 3,3′-diaminobenzidine (DAB) for 1 min, and nuclei were counterstained with hematoxylin. Slides were dehydrated in an ethanol gradient, mounted with neutral gum and stored at room temperature for later observation.

### Imaging and data analysis

For each sample, the number of tumor-infiltrating mononuclear cells (TIMCs) and the membrane expression of CTLA-4 in tumor cells or TIMCs was determined by two independent pathologists blinded to the clinical data. The CTLA-4 expression score in tumor cells was determined according to the staining intensity and the percentage of positively stained cells. The staining intensity was scored as follows: 0 (none), 1 (weak), 2 (moderate) and 3 (strong). The proportion of positively stained cells was scored as: 0 (0%–5%, negative), 1 (5%–25%, sporadic), 2 (25%–50%, focal) and 3 (>50%, diffuse). The final score was calculated as the sum of the percentage and intensity scores, and ranged from 0 to 6. CTLA-4 expression was defined as: “−” (negative; score 0–1), “+ ” (weakly positive; score 2–3), “++” (moderate positive; score 4–5), and “+++” (strongly positive; score 6). Samples with “−” and “+” were considered to have low CTLA-4 expression, whereas those with “++” and “+++” were considered to have high CTLA-4 expression. The extent of CTLA-4-positive TIMCs was assessed as absent (0), focal (1), mild (2), moderate (3), and severe (4) with scores of 0 or 1 considered negative, and samples with a score of 2–4 considered CTLA-4-positive. Four samples were non-evaluable for the number of TIMCs and extent of CTLA-4 staining of TIMCs.

### Statistical analysis

The primary objective of this study was to correlate the levels of CTLA-4 expression with overall survival (OS) in patients with esophageal cancer. We also carried out an exploratory analysis to correlate CTLA-4 expression with clinicopathological characteristics, which were summarized descriptively. OS was described as the time interval from diagnosis to the date of the death or loss to follow-up. Fisher's exact tests were used to assess the associations of clinicopathological characteristics with CTLA-4-positivity in tumor cells and TIMCs. The Cox regression model was used to assess the association of CTLA-4-positivity and TIMCs with OS in both univariate and multivariate analyses. Hazard ratio (HR) and 95% confidence intervals (CI) were also listed. All statistical analyses were carried out using SPSS 19.0 (SPSS, Chicago, IL, USA) and a *P*-value (two-sided) of <0.05 was considered to indicate statistical significance.

## SUPPLEMENTARY TABLES


