# Supplementary material for: Cytotoxic T lymphocyte antigen-4 expression in esophageal carcinoma: implications for prognosis
Source: Oncotarget. 2016 Mar 30;7(18):26670–9. doi: 10.18632/oncotarget.8476 (PMC5042006; doi:10.18632/oncotarget.8476)
Supplement: Supplementary file 1 [file oncotarget-07-26670-s001.pdf]

SUPPLEMENTARY TABLES

Supplementary Table S1: Clinicopathological features of the 158 patients with esophageal cancer (at the time of initial diagnosis)

Clinicopathological parameter	*n* (%)
Age (years)	
≤55	82 (51.9)
>55	76 (48.1)
Histological grade	
Well differentiated	43 (27.2)
Moderately differentiated	75 (47.5)
Poorly differentiated	40 (25.3)
Length	
<3 cm	11 (7.0)
3–5cm	82 (51.9)
>5 cm	65 (41.1)
Tumor size	
T1	9 (5.7)
T2	39 (24.7)
T3	107 (67.7)
T4	3 (1.9)
Lymph node metastasis number	
N0	86 (54.4)
N1	31 (19.6)
N2	15 (9.5)
N3	9 (5.7)
N4	7 (4.4)
N5	1 (0.6)
N6	3 (1.9)
N7	1 (0.6)
N8	2 (1.3)
N9	2 (1.3)
N10	1 (0.6)
Distant metastasis	
M0	152 (96.2)
M1	6 (3.8)
Clinical stage	
IA	3 (1.9)

(*Continued*)

**Clinicopathological parameter**	***n* (%)**
IB	5 (3.2)
IIA	43 (27.2)
IIB	46 (29.1)
IIIA	34 (21.5)
IIIB	14 (8.9)
IIIC	7 (4.4)
IV	6 (3.8)
Location	
Upper	12 (7.6)
Middle	98 (62.0)
Lower	48 (30.4)
CTLA-4 expression on tumor cell	
Negative	42 (26.6)
Weak	44 (27.8)
Moderate	38(24.1)
Strong	34(21.5)
Extent of TIMCs	
Absent	4 (2.5)
Focal	12 (7.6)
Mild	65 (41.1)
Moderate	71 (44.9)
Severe	6 (3.8)
CTLA-4 expression in TIMCs	
Absent	23 (14.9)
Focal	42 (27.3)
Mild	53 (34.4)
Moderate	20 (13.0)
Severe	16 (10.4)

Supplementary Table S2: Correlation between CTLA-4 expression on tumor cells and the clinicopathological characteristics of the 158 ESCC cases

Clinicopathologic variables	Number of each group	CTLA-4 expression on tumor		*P*-value
		Low	High	
All cases	158	86	72	
Age (years)				0.636
<56	97	52	45	
≥56	61	34	27	
Sex				0.488
Male	126	68	58	
Female	32	18	14	
Tumor size (cm)				0.527
<5	63	34	29	
≥5	95	52	43	0.358
Histological differentiation				0.765
Well	43	24	19	
Moderate	75	42	33	
Poor	40	20	20	
TNM				0.361
TNM (1+2)	100	56	44	
TNM (3+4)	58	30	28	
Location				0.112
Upper	12	10	12	
Middle	98	51	47	
Lower	48	25	23	
Lymphatic metastasis				0.461
Negative	86	46	40	
Positive	72	40	32	
Distant Metastasis				0.572
No	152	83	69	
Yes	6	3	3	
TIL				0.11
Absent	4	2	2	
Focal	77	33	44	
Moderate	71	47	24	
Marked	6	4	2	

Supplementary Table S3: Correlation between CTLA-4 expression in TIMCs and the clinicopathological characteristics of the 154[a] ESCC

Clinicopathologic variables	Number of each group	CTLA-4 expression in TIMCs		*P*-value
		negative	positive	
All cases	154	65	89	
Age(years)				0.629
<56	79	31	48	
≥56	75	34	41	
Sex				0.222
Male	124	53	71	
Female	30	12	18	
Tumor size (cm)				0.742
<5	62	20	42	
≥5	92	45	47	0.358
Histological differentiation				0.847
Well	43	20	23	
Moderate	73	28	45	
Poor	38	17	21	
TNM				0.180
TNM (1+2)	98	41	57	
TNM (3+4)	56	24	32	
Location				0.096
Upper	12	7	5	
Middle	95	39	56	
Lower	47	19	28	
Lymphatic metastasis				0.332
Negative	84	45	39	
Positive	70	20	50	
Distant Metastasis				0.405
No	148	61	87	
Yes	6	4	2	
TIMCs[a]				0.08
Focal	77	39	38	
Moderate	71	23	48	
Marked	6	3	3	

Supplementary Table S4: Correlation between CTLA-4 expression profile and the clinicopathological characteristics of the 154[a]ESCC

Clinicopathologic variables	Number of each group	CTLA-4 expression profile		*P*-value
		FCEP Group	Other group	
All cases	154	49	105	
Age(years)				0.747
<56	94	29	65	
≥56	60	20	40	
Sex				0.500
Male	124	41	83	
Female	30	8	22	
Tumor size (cm)				0.923
<5	62	20	42	
≥5	92	29	63	0.358
Histological differentiation				0.926
Well	43	13	30	
Moderate	73	23	50	
Poor	38	13	25	
TNM				0.513
TNM (1+2)	98	33	65	
TNM (3+4)	56	16	40	
Location				0.212
Upper	12	6	6	
Middle	95	26	69	
Lower	47	17	30	
Lymphatic metastasis				0.801
Negative	84	26	58	
Positive	70	23	47	
Distant Metastasis				0.329
No	148	46	102	
Yes	6	3	3	
